# Relationships between retinal layer thickness and brain volumes in the UK Biobank cohort

**DOI:** 10.1111/ene.14706

**Published:** 2021-01-20

**Authors:** Sharon Y. L. Chua, Gerassimos Lascaratos, Denize Atan, Bing Zhang, Charles Reisman, Peng T. Khaw, Stephen M. Smith, Paul M. Matthews, Axel Petzold, Nicholas G. Strouthidis, Paul J. Foster, Anthony P. Khawaja, Praveen J. Patel, The UK Biobank Eye, Vision Consortium

**Affiliations:** ^1^ NIHR Biomedical Research Centre Moorfields Eye Hospital NHS Foundation Trust and UCL Institute of Ophthalmology London UK; ^2^ Kings College Hospital London UK; ^3^ Department of Ophthalmology School of Medicine King's College London London UK; ^4^ Bristol Eye Hospital University Hospitals Bristol NHS Foundation Trust Bristol UK; ^5^ Bristol Medical School University of Bristol Bristol UK; ^6^ Department of Psychiatry University of Oxford Oxford UK; ^7^ Topcon Healthcare Solutions, Research and Development Oakland NJ USA; ^8^ Centre for Functional MRI of the Brain (FMRIB) Wellcome Centre for Integrative Neuroimaging Nuffield Department of Clinical Neurosciences University of Oxford Oxford UK; ^9^ Department of Brain Sciences Faculty of Medicine Imperial College London London UK

**Keywords:** brain MRI markers, cognitive impairment, optical coherence tomography, retinal layers, retinal neurodegeneration

## Abstract

**Background and purpose:**

Current methods to diagnose neurodegenerative diseases are costly and invasive. Retinal neuroanatomy may be a biomarker for more neurodegenerative processes and can be quantified in vivo using optical coherence tomography (OCT), which is inexpensive and noninvasive. We examined the association of neuroretinal morphology with brain MRI image‐derived phenotypes (IDPs) in a large cohort of healthy older people.

**Methods:**

UK Biobank participants aged 40 to 69 years old underwent comprehensive examinations including ophthalmic and brain imaging assessments. Macular retinal nerve fibre layer (mRNFL), macular ganglion cell‐inner plexiform layer (mGCIPL), macular ganglion cell complex (mGCC) and total macular thicknesses were obtained from OCT. Magnetic resonance imaging (MRI) IDPs assessed included total brain, grey matter, white matter and hippocampal volume. Multivariable linear regression models were used to evaluate associations between retinal layers thickness and brain MRI IDPs, adjusting for demographic factors and vascular risk factors.

**Results:**

A total of 2131 participants (mean age 55 years; 51% women) with both gradable OCT images and brain imaging assessments were included. In multivariable regression analysis, thinner mGCIPL, mGCC and total macular thickness were all significantly associated with smaller total brain (*p* < 0.001), grey matter and white matter volume (*p* < 0.01), and grey matter volume in the occipital pole (*p* < 0.05). Thinner mGCC and total macular thicknesses were associated with smaller hippocampal volume (*p* < 0.02). No association was found between mRNFL and the MRI IDPs.

**Conclusions:**

Markers of retinal neurodegeneration are associated with smaller brain volumes. Our findings suggest that retinal structure may be a biomarker providing information about important brain structure in healthy older adults.

## Graphical abstract

Thinner inner retinal layers and total macular thickness were associated with smaller brain volumes in a cohort of healthy UK Biobank adults. Our findings suggest that retinal structure may be a biomarker for processes affecting brain structure.

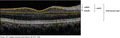

**Abbreviations**
AD: Alzheimer disease
BMI: body mass index
CI: confidence interval
IDP: image‐derived phenotype
IOP: intraocular pressure
IOPcc: corneal‐compensated intraocular pressure
logMAR: logarithm of the minimum angle of resolution
MCI: mild cognitive impairment
mGCC: macular ganglion cell complex
mGCIPL: macular ganglion cell‐inner plexiform layer
mGCL: macular ganglion cell layer
mIPL: macular inner plexiform layer
MRI: magnetic resonance imaging
mRNFL: macular retinal nerve fibre layer
pRNFL: peripapillary retinal nerve fibre layer
SD‐OCT: spectral‐domain optical coherence tomography
TABS: Topcon Advanced Boundary Segmentation

## INTRODUCTION

Neurodegenerative diseases are emerging as the foremost challenge for biomedical science in the 21st century. It has been estimated that 46 million people are living with dementia, and this number is expected to rise to 131 million by 2050 [1, 2]. Prevalence of dementia increases with age, affecting 11%, 32% and 82% of people aged over 65, 75 and 85 years, respectively [3]. Some projections suggest that, due to population aging, the prevalence of Alzheimer disease (AD), the most common form of dementia, may triple by 2050 [2, 3]. Studies have reported age‐related decreases in global and regional brain changes measured by magnetic resonance imaging (MRI) [4]. Brain volume declines with age at a rate of around 5% per decade after age 40, and the rate of decline accelerates after 70 years of age [5, 6]. Brain MRI is accurate and sensitive in detecting structural variations in the brain, some of which are associated with neurodegeneration, but is relatively costly and time consuming [7]. Simpler, resource saving, reproducible biomarkers would offer the prospect of enabling earlier diagnosis of dementia.

The retina and optic nerve share their embryological origin with the brain, and are widely regarded as part of the central nervous system [8]. Retinal microvasculature and neuronal components offer a unique window on tissues that are closely allied to intracranial structures [9]. Consequently, the eye is vulnerable to similar processes that are associated with neurodegenerative diseases [8]. Spectral‐domain optical coherence tomography (SD‐OCT) is an in vivo imaging tool that allows noninvasive, high‐resolution examination of retinal structure [10]. Automated segmentation techniques now make quantitative assessment of retinal sublayers a viable proposition [11]. This offers the prospect of retinal imaging contributing to the diagnosis and monitoring of diseases characterised by structural changes in the brain.

There is a well‐recognised link between dementia and degenerative changes in the retina and optic nerve [12]. Both histopathological and clinical studies have shown that evidence of retinal ganglion cell degeneration in AD patients[13, 14, 15, 16], but only the Rotterdam Study has evaluated whether retinal degeneration may be a marker for subclinical brain disease [17]. To date, most interest has focused on the thickness of the peripapillary retinal nerve fibre layer (pRNFL) as a potential biomarker for dementia [18]. Few studies have examined the relationship between retinal sublayer thickness and cerebral atrophy on MRI [17, 19]. In this UK Biobank study, we aimed to investigate the association between inner retinal layer thicknesses and regional brain volumes in a healthy population to better understand how accessible and affordable eye measurement may contribute as a biomarker for brain structure.

## METHODS

### Study population

UK Biobank is a very large community‐based cohort of UK residents registered with the National Health Service and aged 40 to 69 years at enrolment. Baseline examinations were carried out between 2006 and 2010 at 22 study assessment centres. The North West Multicentre Research Ethics Committee approved the study in accordance with the principles of the Declaration of Helsinki. The overall study protocol (http://www.ukbiobank.ac.uk/resources/) and protocols for individual tests (http://biobank.ctsu.ox.ac.uk/crystal/docs.cgi) are available online. In brief, participants answered a wide‐ranging touch‐screen questionnaire covering demographic, socioeconomic and lifestyle information. Physical measures included blood pressure, height and weight. Body mass index (BMI) was defined as weight divided by height squared.

### Ocular measurements

In late 2009, eye measurements were conducted in six assessment centres by trained staff following standard operating procedures; detailed methods have been published [20, 21]. Visual acuity and intraocular pressure (IOP) were measured. Corneal‐compensated IOP (IOP_cc_) was measured with the ocular response analyser (Reichert, Philadelphia, PA, USA) to examine the influence that corneal biomechanical characteristics might have on IOP measures [20, 22].

### Spectral‐domain optical coherence tomography

Spectral‐domain optical coherence tomography imaging was performed using the Topcon 3D OCT‐1000 Mk2 (Topcon, Tokyo, Japan). Image acquisition was performed under mesopic conditions, without pupillary dilation using the three‐dimensional macular volume scan (512 horizontal A scans per B scan; 128 B scans in a 6 × 6 mm^2^ raster pattern). The Topcon Advanced Boundary Segmentation (TABS) algorithm was used for automated segmentation [23]. Retinal layers were labelled as macular retinal nerve fibre layer (mRNFL), macular ganglion cell‐inner plexiform layer (mGCIPL), macular ganglion cell complex (mGCC) and total macular thickness. Thickness of the mGCIPL includes the macular ganglion cell layer (mGCL) and macular inner plexiform layer (mIPL), whereas mGCC includes both mRNFL and mGCIPL. We followed the Advised Protocol for OCT Study Terminology and Elements (APOSTEL) guidelines, except with respect to the total macular thickness, which we define as the thickness from the inner limiting membrane to the retinal pigment epithelium [24]. mRNFL is composed of axons of the retinal ganglion cell, whereas mGCL is composed of ganglion cell bodies, and mIPL is composed of retinal ganglion cell dendrites. We used the average thickness of the retinal layers in the macula across nine retinal subfields in a 6‐mm‐diameter circle centred at the true fovea location, as derived from the ETDRS (Early Treatment Diabetic Retinopathy Study) [25]. We have added the prefix “m” to the sublayer abbreviations to denote as macular measures, as some SD‐OCT metrics can be derived from images centred on the optic disc.

### MRI Brain Image‐Derived Phenotypes (IDPs)

MRI imaging protocols were designed by the UK Biobank Imaging Working Group (http://www.ukbiobank.ac.uk/expert‐working‐groups). Details of the image acquisition and processing are available on the UK Biobank website in the brain scan protocol (http://biobank.ctsu.ox.ac.uk/crystal/refer.cgi?id=2367) and brain imaging documentation (http://biobank.ctsu.ox.ac.uk/crystal/docs/brain_mri.pdf). Briefly, brain imaging was carried out using a single standard Siemens Skyra 3T scanner with a 32‐channel radio‐frequency (RF) receive head coil. Key acquisition parameters for each modality are summarised in a prior publication [26]. The T1‐weighted scans were acquired using three‐dimensional magnetization‐prepared rapid gradient‐echo (MPRAGE) (resolution 1 mm^3^ isotropic voxels) and analysed with the Functional Magnetic Resonance Imaging of the Brain (FMRIB) Software Library (FSL) (http://fsl.fmrib.ox.ac.uk/fsl). Further details on MRI acquisition and analysis have been reported elsewhere [26, 27]. Brain images were analysed to derive multiple distinct individual measures of brain structure and function, known as brain image‐derived phenotypes (IDPs) [26]. Total brain volume was defined as the sum of grey matter volume and white matter volume. Grey matter volume in the frontal, temporal and occipital poles and hippocampal volume were averaged from the right and left volumes. Total brain, grey matter and white matter volumes were normalised for head size. Grey‐matter volume in the frontal, temporal and occipital poles (V1/V2) and hippocampus volume were normalised for head size by multiplying the individual brain IDPs by the volumetric scaling from T1 head image to standard space. Brain IDPs were normalised for head size, which is a close proxy for intracranial volume [28]. Normalised brain IDPs were termed as total brain, grey matter, white matter and hippocampal normalised.

### Inclusion and exclusion criteria

To ensure the accuracy of retinal thickness assessment, we have excluded OCT scans of poor quality consistent with the OSCAR IB’’ [(O)= obvious problems including violation of the protocol; (S) poor signal strength defined as ,15 dB; (C) wrong centration of scan; (A) algorithm failure; (R) retinal pathology other than MS related; (I) illumination; and (B) beam placement]. (OSCAR‐IB) criteria [29]. In general, the OSCAR‐IB criteria requires good quality OCT scans and signal strength with no visible retinal pathology. Among those with SD‐OCT data, we excluded participants who withdrew their consent, had poor SD‐OCT signal strength, had an image quality score <45, had poor centration certainty or had poor segmentation certainty using TABS software [30, 31]. Participants with the following conditions were also excluded from the study: visual acuity worse than 0.5 logarithm of the minimum angle of resolution (logMAR) (approximately 6/18 on Snellen chart), IOP_cc_ of <6 mm Hg or >24 mm Hg [32], self‐reported ocular disorders or diseases, self‐reported diabetes or self‐reported neurodegenerative disease. This group of participants were excluded from the study because of the well‐recognised impact these conditions have on retinal layer thickness [33, 34, 35].

### Statistical analysis

The present analysis was based on cross‐sectional data. For this analysis, if both eyes of a patient were eligible for inclusion, one eye was randomly selected using Stata software (version 16; StataCorp, College Station, TX, USA). The *z* scores of the four retinal sublayer thickness and brain MRI markers were calculated by subtracting the mean value from the value of the observation and dividing by the standard deviation. We examined the association of retinal sublayer thickness (explanatory or independent variable) with brain MRI markers (dependent variable) using two multivariable linear regression models: Model 1 adjusted for age, age squared and sex; and Model 2 additionally adjusted for race, education, BMI, mean arterial blood pressure and smoking status. Cardiovascular risk factors including BMI, mean arterial blood pressure and smoking status were adjusted for in the multivariable models in view of their relationship with brain MRI markers [36, 37, 38] and retinal layers[39].We minimised the effect of age on cerebral atrophy by adjusting for age and age squared. In sensitivity analysis, we additionally adjusted for total grey matter normalised when the outcome was hippocampal normalised. The β coefficients represent standardised mean difference in *z* scores of total brain, grey or white matter and hippocampal normalised per SD decrease in retinal sublayer thickness. We applied linear transformation and have used *z* scores as standardizing scores to facilitate the interpretation of the value of the individual brain IDPs.

## RESULTS

**FIGURE 1:**
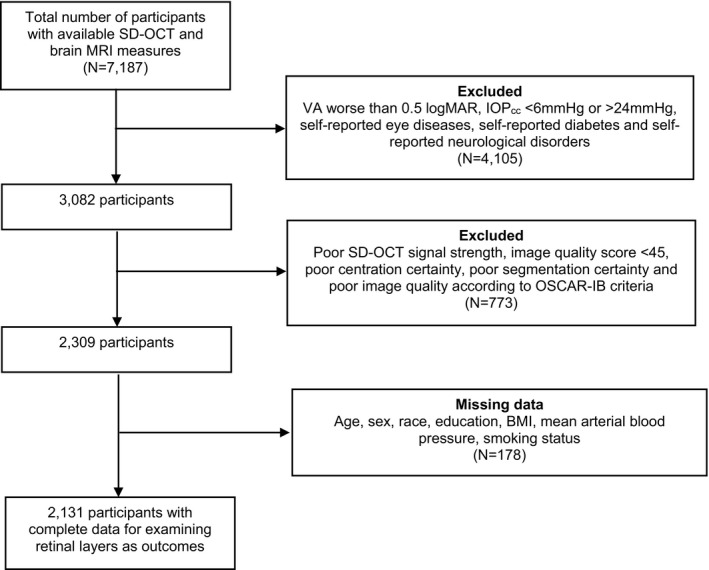
Flowchart of participants included in the study. BMI, body mass index; IOPcc, corneal‐compensated intraocular pressure; logMAR, logarithm of the minimum angle of resolution; MRI, magnetic resonance imaging; SD‐OCT, spectral‐domain optical coherence tomography; VA, visual acuity.

There were 7187 participants with data on SD‐OCT macular imaging and brain MRI scans. Of these, 4105 people with visual acuity worse than 0.5 logMAR (equivalent to 6/18 on Snellen chart), IOP_cc_ of <6 mm Hg or >24 mm Hg, self‐reported eye diseases, diabetes and neurological disorders were excluded. Of the remaining 3082, 773 participants with poor SD‐OCT image quality were excluded, and 178 participants were further excluded due to missing data for covariables. There were complete data (age, sex, race, education, BMI, mean arterial blood pressure and smoking) for 2131 of these participants (Figure 1).

The characteristics of the study population are shown in Table 1. The mean age was 54.6 years (SD = 7.4), and 1084 (51%) of the participants were female. The overall mean (SD) of the different retinal layers was: mRNFL (29.2 µm [SD, 4.6 µm]), mGCIPL (74.1 µm [5.9 µm]), mGCC (103.3 µm [8.0 µm]) and total macular thickness (278.7 µm [12.7 µm]). Table 2 shows the multivariable analysis of retinal sublayer thickness with total brain, grey matter, white matter and hippocampal normalised after adjusting for age, age squared and sex. In general, thinner mRNFL, mGCIPL, mGCC and total macular thickness were all significantly associated with smaller total brain, grey matter and white matter normalised (except the association between mRNFL and total white matter normalised). Reduced mRNFL, mGCC and total macular thickness were associated with smaller hippocampal normalised, but the associations were not significant (*p* > 0.05) when we additionally adjusted for total grey matter normalised.

**TABLE 1:** Characteristics of the study population (*n* = 2131)

Characteristic	*N*	Mean ± SD^a^	%
Age, years	2131	54.6 ± 7.4	
Sex, %			
Male	1047		49.1
Female	1084		50.9
Race, %			
White	2063		96.8
Non‐White	68		3.2
Education level, %			
O level and less	600		28.2
Professional qualification or A level	553		25.9
Degree and above	978		45.9
Body mass index, kg/m^2^	2131	26.7 ± 4.2	
Smoking status, %			
Never	1370		64.3
Former	640		30.0
Current	121		5.7
Mean arterial blood pressure, mm Hg	2131	100.7 ± 12.1	

^a^ Mean (SD) for continuous variables and percentages for categorical variables.

**TABLE 2:** Age‐ and sex‐adjusted estimated change in brain MRI markers per standard deviation decrease in average mRNFL, mGCIPL, mGCC and total macular thickness

	mRNFL thickness			mGCIPL thickness			mGCC thickness			Total macular thickness		
	β	95% CI	*p* value	β	95% CI	*p* value	β	95% CI	*p* value	β	95% CI	*p* value
Total brain normalised	−0.04	(−0.07, −0.002)	**0.04**	−0.08	(−0.12, −0.05)	**<0.001**	−0.08	(−0.12, −0.05)	**<0.001**	−0.08	(−0.12, −0.05)	**<0.001**
Grey matter normalised												
Total normalised	−0.04	(−0.07, −0.005)	**0.022**	−0.05	(−0.08, −0.01)	**0.007**	−0.06	(−0.09, −0.02)	**0.001**	−0.06	(−0.09, −0.03)	**<0.001**
Frontal pole	−0.02	(−0.06, 0.01)	0.19	−0.04	(−0.07, 0.0008)	0.06	−0.04	(−0.08, −0.004)	**0.030**	−0.04	(−0.08, −0.003)	**0.034**
Temporal pole	0.002	(−0.04, 0.04)	0.92	−0.03	(−0.07, 0.01)	0.21	−0.02	(−0.06, 0.02)	0.39	−0.04	(−0.08, 0.006)	0.09
Occipital pole	−0.02	(−0.06, 0.02)	0.42	−0.06	(−0.10, −0.02)	**0.004**	−0.06	(−0.10, −0.01)	**0.009**	−0.05	(−0.09, −0.009)	**0.017**
White matter volume												
Total normalised	−0.02	(−0.06, 0.02)	0.28	−0.09	(−0.13, −0.05)	**<0.001**	−0.08	(−0.12, −0.04)	**<0.001**	−0.07	(−0.11, −0.03)	**0.001**
Hippocampal normalised	−0.04	(−0.08, −0.004)	**0.030**	−0.03	(−0.07, 0.005)	0.09	−0.05	(−0.09, −0.01)	**0.012**	−0.05	(−0.09, −0.01)	**0.008**

Note

Values represent standardised mean difference in *z* scores of total brain, grey or white matter, and hippocampal normalised per SD decrease in retinal sublayer thickness. Values are adjusted for age, age squared and sex. Bold values denote statistical significance at the *p* < 0.05 level.

Abbreviations: mGCC, macular ganglion cell complex; mGCIPL, macular ganglion cell‐inner plexiform layer; mRNFL, macular retinal nerve fibre layer.

Table 3 shows the multivariable analysis of retinal sublayer thickness with MRI IDPs after adjusting for age, age squared, sex, race, education, BMI, mean arterial blood pressure and smoking status. Thinner mGCIPL, mGCC, and total macular thickness were all significantly associated with smaller total brain normalised, standardised mean difference (95% CI) per SD decrease in retinal sublayer thickness, respectively: −0.08 (95% confidence interval [CI]: −0.12, −0.05; *p* < 0.001), −0.08 (95% CI: −0.12, −0.04; *p* < 0.001) and −0.08 (95% CI: −0.11, −0.04; *p* < 0.001). Reduced mGCIPL, mGCC and total macular thickness were significantly associated with grey matter normalised, standardised mean difference (95% CI) per SD decrease in retinal sublayer thickness, respectively: −0.04 (95% CI: −0.08, −0.01; *p* = 0.009), −0.05 (95% CI: −0.08, −0.02; *p* = 0.003) and −0.06 (95% CI: −0.09, −0.02; *p* = 0.001). Thinner mGCIPL, mGCC and total macular thickness were also significantly associated with white matter normalised, standardised mean difference (95% CI) per SD decrease in retinal sublayer thickness, respectively: −0.09 (95% CI: −0.13, −0.05; *p* < 0.001), −0.08 (95% CI: −0.12, −0.04; *p* < 0.001) and −0.07 (95% CI: −0.11, −0.03; *p* = 0.001). Reduced mGCC thickness was associated with smaller grey matter normalised in the frontal pole, standardised mean difference (95% CI) per SD decrease in mGCC thickness: −0.04 (95% CI: −0.08, −0.002; *p* = 0.038). Thinner mGCIPL, mGCC and total macular thickness were associated with smaller grey matter normalised in the occipital pole, standardised mean difference (95% CI) per SD decrease in retinal sublayer thickness, respectively: −0.06 (95% CI: −0.10, −0.02; *p* = 0.003), −0.06 (95% CI: −0.10, −0.02; *p* = 0.006) and −0.05 (95% CI: −0.09, −0.008; *p* = 0.020). Thinner mGCC and total macular thickness were associated with smaller hippocampal normalised, standardised mean difference (95% CI) per SD decrease in retinal sublayer thickness, respectively: −0.05 (95% CI: −0.09, −0.008; *p* = 0.018), and −0.05 (95% CI: −0.09, −0.01; *p* = 0.010) but were no longer significant (*p* > 0.05) when we additionally adjusted for total grey matter normalised. There were no significant associations between mRNFL thickness and the brain MRI measures. Additional adjustment for IOP_cc_, the sole modifiable risk factor for glaucoma, did not meaningfully change the effect estimates between retinal sublayer thicknesses and brain MRI markers.

**TABLE 3:** Multivariable adjusted estimated change in brain MRI markers per standard deviation decrease in average mRNFL, mGCIPL, mGCC and total macular thickness

	mRNFL thickness			mGCIPL thickness			mGCC thickness			Total macular thickness		
	β	95% CI	*p* value	β	95% CI	*p* value	β	95% CI	*p* value	β	95% CI	*p* value
Total brain normalised	−0.03	(−0.07, 0.004)	0.08	−0.08	(−0.12, −0.05)	**<0.001**	−0.08	(−0.12, −0.04)	**<0.001**	−0.08	(−0.11, −0.04)	**<0.001**
Grey matter normalised												
Total normalised	−0.03	(−0.06, 0.002)	0.06	−0.04	(−0.08, −0.01)	**0.009**	−0.05	(−0.08, −0.02)	**0.003**	−0.06	(−0.09, −0.02)	**0.001**
Frontal pole	−0.02	(−0.06, 0.01)	0.24	−0.04	(−0.07, 0.001)	0.06	−0.04	(−0.08, −0.002)	**0.038**	−0.04	(−0.08, 0.0001)	0.05
Temporal pole	0.005	(−0.04, 0.05)	0.80	−0.02	(−0.07, 0.02)	0.23	−0.02	(−0.06, 0.03)	0.46	−0.03	(−0.07, 0.007)	0.11
Occipital pole	−0.02	(−0.06, 0.02)	0.37	−0.06	(−0.10, −0.02)	**0.003**	−0.06	(−0.10, −0.02)	**0.006**	−0.05	(−0.09, −0.008)	**0.020**
White matter normalised												
Total normalised	−0.02	(−0.06, 0.02)	0.32	−0.09	(−0.13, −0.05)	**<0.001**	−0.08	(−0.12, −0.04)	**<0.001**	−0.07	(−0.11, −0.03)	**0.001**
Hippocampal normalised	−0.04	(−0.08, 0.0002)	0.05	−0.03	(−0.07, 0.006)	0.09	−0.05	(−0.09, −0.008)	**0.018**	−0.05	(−0.09, −0.01)	**0.010**

Note

Values represent standardised mean difference in *z* scores of total brain, grey or white matter and hippocampal normalised per SD decrease in retinal sublayer thickness. Values are adjusted for age, age squared, sex, race, education, body mass index, mean arterial blood pressure and smoking. Bold values denote statistical significance at the *p* < 0.05 level.

Abbreviations: mGCC, macular ganglion cell complex; mGCIPL, macular ganglion cell‐inner plexiform layer; mRNFL, macular retinal nerve fibre layer.

## DISCUSSION

In this study, we identified that thinner mGCIPL, mGCC and total macular thickness were associated with smaller total brain normalised, grey and white matter normalised, and grey matter normalised in the occipital pole. Reduced mGCC was associated with smaller grey matter normalised in the frontal pole, whereas mRNFL thickness was not associated with the MRI IDPs. This suggests that retinal thickness measures provide not only useful information on brain function, but also on anatomically relevant brain structures.

Cognitive impairment in dementia is characterised by cerebral atrophy. Our study has shown that thinner mGCIPL, mGCC and total macular thicknesses were associated with smaller total brain, grey and white matter normalised. In addition, because of the age‐related effects on cerebral atrophy, we adjusted for age and age squared to minimise the possibility of confounding by age. Our data suggest that neuronal damage may occur in the retina and throughout the brain. Our result was consistent with a report from the Rotterdam Study Group that reported thinner inner retinal layers were associated with total brain, grey and white matter volumes [17]. Our findings showed that thinner mGCIPL, mGCC and total macular thicknesses were associated with reduced grey‐matter normalised in the occipital pole. It is well known that atrophy of the occipital lobe, particularly V1/V2, is known to cause inner retinal layer atrophy by retrograde transsynaptic axonal degeneration. In agreement with our findings, Ong et al. reported that thinner mGCIPL was associated with smaller grey matter volume in the occipital lobe [19]. These data are also consistent with a number of studies in control subjects and patients with multiple sclerosis [40, 41, 42]. The association of inner retinal layer atrophy was strongest for V1 and V2 using validated segmentation software. Taken together, the published data suggest a strong association between pathology and atrophy in these areas. The question becomes more complex for impaired higher visual function. AD pathology of the lateral occipital cortices and parietal lobes are not known to be associated with atrophy of inner retinal layers in the macula. Therefore, a limitation of the study is that impaired visual perception in AD is unlikely to be captured by atrophy of the mGCC/mGCIPL.

Hippocampal atrophy is a major feature of AD and a diagnostic marker for AD at the mild cognitive impairment stage [43]. Histological studies have shown considerable neuronal loss in the hippocampus at onset of symptoms [44]. In agreement with histological findings, longitudinal studies reported accelerated hippocampal volume loss in AD [45, 46, 47, 48] and mild cognitive impairment (MCI) [49, 50]. In addition to smaller hippocampal volumes observed in patients with cognitive impairment, evidence also suggests that the inhibition of adult hippocampal neurogenesis leads to memory impairments [51, 52], whereas enhancing neurogenesis improves memory performance [53, 54]. Given that hippocampal atrophy is a prominent feature of AD and MCI, our findings that, in an adult population, thinner mGCC and total macular thicknesses were associated with smaller hippocampal normalised, suggests that common mechanisms may lead to hippocampal and retinal atrophy. Additionally, we examined the association between total macular thickness and brain MRI, and observed that thinner total macular thickness was associated with smaller brain MRI structural measures. Because the presence of imaging artefacts may decrease the accuracy of specific retinal thickness measurements, measurement of total macular thickness is more accurate compared to specific sublayer retinal thickness [55]. In line with our findings, the Rotterdam Study reported that thinner inner retinal layers were associated with smaller hippocampal volumes, although grey volume was not adjusted in the multivariable models [17].

Nonetheless, in a sensitivity analysis, we did not find an association between the inner retinal layers or total macular thickness and hippocampal normalised after the additional adjustment of total grey matter volume. This suggests that, at least among the healthy UK Biobank participants, the relationship between thinner retinal measures and smaller hippocampal volumes is explicable as a consequence of smaller total grey matter volume [56], which could be a consequence of either global neurodegeneration or differences in neurodevelopment. Measurement of relative rates of volume loss over time in the same individuals will disambiguate this question.

Previous studies by Blanks et al. [13] and Hinton et al. [14] reported that patients with AD have apparent histological signs of retinal ganglion cell and mRNFL loss compared to controls, consistent with accelerated neurodegeneration. Clinic‐based studies have demonstrated that patients with AD or mild cognitive impairment have reduced pRNFL [34], mGCIPL [57], mGCC [58] and total macular thickness [59, 60] compared to controls. AD is associated with cerebral atrophy on high‐resolution MRI [43]. Although our findings suggest that inner retinal and total macular thickness measurements may be a biomarker for brain structure, further work would be needed to examine the utility of retinal parameters as a diagnostic or screening tool for diseases that affect brain structure.

We did not identify significant associations between mRNFL thickness and brain MRI structural measures. However, pRNFL was not measured in our study. Thinner pRNFL was associated with grey matter volume in the temporal lobe in a Singapore study [19] and thinner pRNFL was associated with smaller grey matter and white matter volumes in the Rotterdam Study [17]. As the mRNFL axon bundles that arise from retinal ganglion cells converge toward the optic disc, mRNFL in the macula is much thinner compared to pRNFL (and vice versa for mGCIPL) [61], which may explain the lack of association between mRNFL thickness and brain MRI measures in our study. In addition, reduction in dendritic complexity and area occurs prior to retinal ganglion cell death and loss [62], which suggests that mGCIPL may be more sensitive at detecting neurodegenerative damage compared to mRNFL.

The strengths of our study include its large sample, the quantitative assessment of retinal sublayers on SD‐OCT and the brain structures on MRI. Limitations of the study include the UK Biobank is a volunteer cohort, and participants are likely to be healthier than the general population. In addition, a large number of the participants with eye data did not yet have data on brain structural MRI measures. The self‐reported nature of glaucoma, ocular disorders, diabetes or neurodegenerative disease also could result in misclassification bias. However, as both the retinal structures measured with SD‐OCT and brain MRI structures are objective measures, the latter is more likely to be nondifferential misclassification and bias the effect estimates toward the null. The cross‐sectional design of our study limits the ability to determine the cause and effect of the relationship between retinal structures and brain MRI markers. Among healthy older adults (i.e., our cohort), there is extreme variability in the relationship between hippocampal size and memory [63]. Our study may not be able to identify pathological trends, but rather provides information on the trends to be expected in a healthy, older population.

In conclusion, thinner mGCIPL, mGCC and total macular thickness are associated with smaller total brain, grey matter and white matter normalised in a cohort of healthy adults. Our findings support the concept that retinal layer thickness is a biomarker for processes affecting central nervous system structure. Further research examining the utility of retinal measures as an affordable, noninvasive investigation of neurological conditions is warranted.

## CONFLICT OF INTEREST

Charles Reisman reports employment by Topcon Healthcare Solutions, Inc. outside the submitted work. Paul J. Foster reports personal fees from Allergan, Carl Zeiss, Google/DeepMind and Santen, a grant from Alcon, outside the submitted work. Praveen J. Patel reports grants from Topcon Inc., outside the submitted work. Anthony P. Khawaja reports personal fees from Aerie, Allergan, Google Health, Novartis, Thea and Santen, all outside the submitted work. Sharon Y. L. Chua, Axel Petzold, Nicholas G. Strouthidis, Gerassimos Lascaratos, Denize Atan, Bing Zhang, Stephen M. Smith, Paul M. Matthews and Peng T. Khaw declare no competing interests.

## AUTHORS' CONTRIBUTIONS

Peng T. Khaw and Paul J. Foster contributed to the conception and design of the study. Sharon Y. L. Chua and Paul J. Foster contributed to the data analyses, data interpretation and wrote the draft of the manuscript. All authors reviewed the results, and read and critically revised the manuscript. All authors approved the final manuscript.

## ETHICAL APPROVAL

The North West Multicenter Research Ethics Committee approved the study (reference no. 06/MRE08/65), in accordance with the tenets of the Declaration of Helsinki. Detailed information about the study is available at the UK Biobank website (www.ukbiobank.ac.uk)

## Data Availability

Researchers wishing to access UK Biobank data can register and apply at https://www.ukbiobank.ac.uk/.
